# Flexibility, Resistance, Aerobic, Movement Execution (FRAME) training program to improve gait capacity in adults with Hereditary Spastic Paraplegia: protocol for a single-cohort feasibility trial

**DOI:** 10.3389/fneur.2025.1441512

**Published:** 2025-02-18

**Authors:** Leonardo Boccuni, Marco Bortolini, Cristina Stefan, Valentina Dal Molin, Giacomo Dalla Valle, Andrea Martinuzzi

**Affiliations:** Scientific Institute, IRCCS E. Medea, Department of Conegliano, Treviso, Italy

**Keywords:** hereditary spastic paraplegia, neurorehabilitation, gait training, spasticity, core stability, balance, motor control, aerobic training

## Abstract

**Background:**

Hereditary Spastic Paraplegia (HSP) is a heterogeneous group of inherited neurological disorders characterized by progressive weakness and spasticity in the lower limbs, significantly affecting gait capacity (endurance and speed). Although specific interventions have been already investigated, there is currently a lack of comprehensive, structured neurorehabilitation programs to improve gait capacity in adults with HSP. Thus, this protocol aims to explore the feasibility and effectiveness of a composite training targeting flexibility, muscle strength, motor control, balance, and aerobic capacity.

**Methods:**

20 adults diagnosed with HSP will participate in 10 to 16 therapist-guided sessions (intervention), lasting 60 to 120 minutes each, occurring once or twice weekly based on individual preferences. Depending on the number and frequency of sessions, the intervention period may vary in between five to 10 weeks. Upon completion, participants will receive a transfer package (manual, video tutorials) to stimulate long-term exercise at home. Assessments will take place before intervention (T0), at the end of the intervention (T1), and 12 weeks post-T1 (T2). Primary outcomes will focus on feasibility (recruitment, retention, adherence, absence of adverse events, and patient's satisfaction). Secondary outcomes will evaluate improvements in gait capacity and specific contributing factors such as reduced spasticity, increased muscle strength, and improved balance.

**Relevance:**

The significance of this protocol is to provide valuable insights for clinicians regarding the feasibility and potential efficacy of a comprehensive, clinical-oriented program to improve gait capacity in adults with HSP, and inform future translational research studies in the field.

**Clinical trial registration:**

ClinicalTrials.gov, identifier NCT06742697.

## Introduction

Hereditary Spastic Paraplegia (HSP) represent a group of heterogeneous genetic neurodegenerative diseases affecting the corticospinal tract and the dorsal column in a length-dependent fashion, with a combined prevalence of two to five cases each 100,000 individuals worldwide (1). The activity that is most commonly affected is gait, typically due to the progressive development of lower limb weakness and spasticity (2). In addition to that, other disorders include ataxia, sensory deficits, cognitive impairments, urinary symptoms, dysarthria, hyperreflexia, and seizures (2).

Currently, there is no disease-modifying therapy for HSP, thus symptomatic treatment is the cornerstone of HSP management (2–4). Besides medical therapies (oral antispasmodics, botulinum toxin injections) neurorehabilitation may help improve gait function, by increasing range of motion, strength, balance, and endurance (4). However, current literature is scant and heterogeneous, based on small samples and often lacking a control intervention, thus hampering the definition of best treatment approaches. One systematic and one narrative review reported low/medium quality evidence for functional electrical stimulation, robotic-assisted gait training, hydrotherapy, and heating to improve gait in patients with HSP, by reducing spasticity and strengthening lower limb muscles (3, 4).

The typical approach is investigating the effect of a single therapeutic strategy to remedy a multifaceted disorder. Alternatively, the focus of research may be to investigate the effect of a comprehensive treatment, including several therapeutic ingredients and adaptable to specific patient's needs, capabilities, and preferences (5–7). For instance, Constraint-Induced Movement Therapy is considered the most effective intervention to improve upper limb motor impairment post-stroke (8); it is a composite intervention based on motor learning principles, whose ingredients include intensive graded task practice, restraint of unaffected arm, and a transfer package for long-term maintenance of functional gains (9). Similarly, composite training may be effective to improve gait for patients with HSP and represent an appropriate strategy to tackle such multifaceted disorders. Moreover, this approach better reflects current practice in the real world, whereas the provision of a single technique in rehabilitation is seldom applicable. To date, only a case series with two participants investigated intensive composite neurorehabilitation on patients with HSP, showing promising yet preliminary findings that need to be confirmed in larger cohort studies (10).

When looking at common upper motor neuron syndromes such as stroke, there is more compelling evidence that may help identify potentially effective treatments for HSP, to be included as active ingredients of the intervention. For instance, electrical stimulation has shown consistent effectiveness on reducing lower limb spasticity, likely by normalizing abnormal spinal circuitry (increase of post-activation depression) (11, 12). Furthermore, mobilization with movement showed superior outcomes than static stretching not only on improving passive range of motion, but also balance, gait speed and cadence (13). When looking at active exercises, trunk training in the form of core stability and instability resistance training has shown positive outcomes on trunk balance and gait (14). Regarding gait training, both overground and speed dependent treadmill gait training showed consistent effectiveness on gait outcomes (maximum gait speed and step width) (15, 16). Other emerging interventions are high intensity interval training (HIIT) and home-based training. Compared with moderate intensity aerobic training, HIIT is superior in terms of cardiovascular benefits (increased endurance), and more acceptable by patients (lower perceived effort) (17). Finally, home-based training has shown functional and cost benefits, particularly relevant for lifelong conditions for which therapist-based training is not always feasible (18).

Therefore, the objective of the proposed single-cohort, uncontrolled pilot trial is to investigate the feasibility and effectiveness of a composite neurorehabilitation program to improve gait function in patients with HSP. The main outcome will be feasibility in terms of recruitment, retention, adherence, safety, and patient's satisfaction of the overall intervention and for each item separately. Secondary outcomes will investigate improvements in gait capacity, here defined as collective term for gait endurance and gait speed, and factors that may have contributed to functional gains, such as normalized muscle tone and improvements for strength, balance, and cardiovascular status.

## Methods: participants, intervention, outcomes, and study timeline

The protocol has been developed according to SPIRIT 2013 guidelines (19, 20). Information related to trial registration are reported in Table 1.

**Table 1:** SPIRIT WHO trial registration data set.

**Data category**	**Information**
Primary registry and trial identifying number	ClinicalTrials.gov (NCT06742697)
Date of registration in primary registry	18/12/2024
Secondary identifying numbers	1,130
Source(s) of monetary or material support	Italian Ministry of Health (Ricerca Corrente 2022-2024)
Primary sponsor	Scientific Institute, IRCCS Eugenio Medea, Department of Conegliano, Treviso, Italy
Contact for public or scientific queries	Leonardo Boccuni (leonardo.boccuni@lanostrafamiglia.it)
Title	Flexibility, Resistance, Aerobic, Movement Execution (FRAME) training program to improve gait capacity in adults with Hereditary Spastic Paraparesis: protocol for a single-cohort feasibility trial.
Countries of recruitment	Italy
Health condition(s) or problem(s) studied	Hereditary Spastic Paraparesis
Intervention(s)	Neurorehabilitation to improve gait speed and endurance
Key inclusion and exclusion criteria	Inclusion: adults with Hereditary Spastic Paraplegia. Exclusion: contraindications to physical exercise.
Study type	Interventional, prospective, single-cohort, pilot feasibility trial
Date of first enrolment	23/12/2024
Target sample size	20
Recruitment status	Recruiting
Primary outcome(s)	Feasibility
Key secondary outcomes	Improvements in gait capacity (6 Minute Walking Test)

All study procedures will be conducted at the IRCCS Eugenio Medea (Italy). Both clinical assessments and therapist-guided sessions will be administered by experienced physiotherapists specialized in neurorehabilitation of patients affected by HSP. Eligibility criteria are as follows: adults (≥18 years of age) diagnosed with Hereditary Spastic Paraplegia; presence of any lower limb impairment affecting gait, such as muscle weakness, spasticity, or balance deficits; ability to walk without physical assistance of another person, as defined by a Functional Ambulation Category of 3 or higher (21, 22); able to understand simple instructions, understand the purpose of the intervention, willing to participate and agree to undertake at least 10 treatment sessions, and able to provide informed consent. Patients will be excluded if they receive botulinum toxin or surgery to remedy spasticity in the 6 months before enrolment or at any time during the intervention period; a physician will screen for contraindications to stretching, resistance training, and/or aerobic exercise such as severe musculoskeletal conditions, decompensated heart failure, severe aortic stenosis, uncontrolled arrhythmia, and acute coronary syndromes. Notably, to be representative of real-world heterogeneity in a clinical setting, patients will be selected from multiple genotypes, varying ages and disease duration, different levels of symptoms severity, and to include a rather comparable number of males and females.

In addition to eligibility criteria, participants will be screened for contraindications to peripheral electrical stimulation, namely the presence of pacemaker or any implanted electronic device, stimulation over areas of known or suspected malignancy, recently radiated tissue, tissue infection, wounds, history of seizures, ongoing or suspected pregnancy, recent surgery, bone fracture, osteoporosis, active deep vein thrombosis or thrombophlebitis, and impaired circulation. In case of presence of any of these conditions, patients will still be deemed eligible but will not receive electrical stimulation.

### Intervention

**Figure 1:**
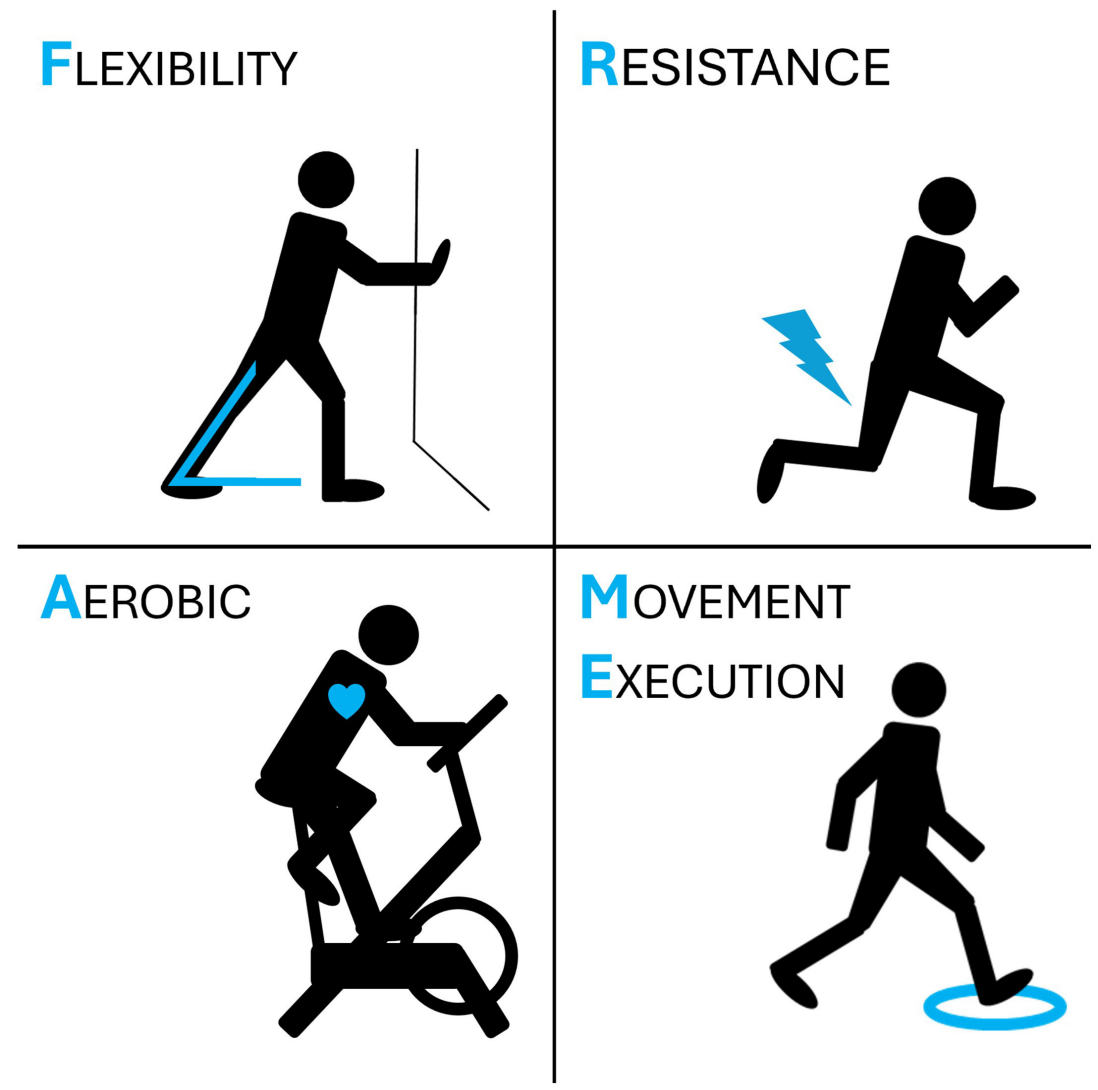
FRAME training to improve gait capacity in adults with HSP.

FRAME (Flexibility, Resistance, Aerobic, Movement Execution) is the acronym indicating a composite neurorehabilitation program to improve gait capacity (Figure 1). FRAME has been designed specifically for this research study and is composed by four main items, here described in the order with which they will be structured within each session:

- Item #1: Flexibility. To reduce muscle tone and increase mobility the therapist will apply a combination of stretching and electrical stimulation. At the level of the ankle joint, mobilization with movement techniques will be preferred over static stretching because of superior efficacy and translation of gains to other functional domains (balance and gait) (13). At the level of adductors and other proximal muscles that typically show stiffness (i.e. rectus femoris, hamstrings), both stretching while lying on a bed and while standing will be considered, based on the patient's capabilities to lengthen the muscle while holding balance. Electrical stimulation will be applied with a portable and programmable electrical stimulation device (GENESY 600, Domino s.r.l., Codognè, Italy, CE marked as neuromuscular stimulator medical device). One to four pairs of self-adhesive electrodes (5 × 5 cm) will be applied over the muscle belly of spastic muscles to deliver biphasic, high-frequency sensory stimulation (100 Hertz, pulse width 200 μs, intensity just below motor threshold) for 30 min (12). Electrical stimulation will be applied during stretching exercises.
- Item #2: Resistance training (and balance). Muscle strength will target muscles that are typically weakened in patients with HSP, i.e., proximal muscles at the level of the hip and the trunk. A recent Cochrane systematic review showed that trunk training has positive effects on trunk control as well as on balance and walking ability (14). Among trunk training interventions, core stability and instability training showed the strongest evidence (14). Compared to strength training in stable conditions (for instance, leg press), training in condition of instability (for instance, performing a squat with feet on foams) has shown higher muscle recruitment, increased coordination, and benefits extending to balance and cognitive functions (23, 24). Therefore, patients with HSP will undertake a protocol of core stability training and lower limb training in conditions of instability, eventually by using foams and/or bosu balls while lying supine, lying prone, sitting, and standing.
- Item #3: Movement Execution (and balance). There are well established motor learning principles that are valid for the general population as well as for neurological patients, for instance graded, repetitive task-specific practice with the provision of adequate feedback (25). When considering gait training, improving motor control initially requires dividing a complex gait pattern into simpler elements, practicing each element consistently until the patient is proficient, and then practicing progressively more complex sequences until the whole gait sequence may be executed automatically. For instance, the first element is standing on both feet, then performing a weight shift over one foot so that the other foot can take a step, then lifting one heel but not the entire foot, then lifting the entire foot and standing on the other, then taking a step with heel contact (but not toe contact), then taking a step with full foot contact of the front foot but with only toe contact of the back foot, and so on. While training on each specific element of gait that is impaired, patients will be challenged with standing balance. When walking, patients will be asked to walk with specific patterns, such as fast walk, slow walk, sidewalk, backward walk, climbing stairs, and so on. Finally, dual task training will be provided to automatize gait while dealing with another motor and/or cognitive task (26).

Item #4: Aerobic training. Among all aerobic training protocols to improve cardiovascular status and endurance, the most feasible and effective seems to be High Intensity Interval Training (HIIT). HIIT consists of short bouts of intense aerobic effort involving major muscle groups, interspersed with periods of rest of comparable duration (either 1:1 ratio, 3:4 ratio, and so on) (27). Compared with moderate intensity aerobic training, HIIT showed higher cardiovascular outcomes, and lower perceived effort (more acceptable by patients) (27). For the present protocol, we will apply bouts of 30 s “all-out”, followed by 30 s of passive rest, repeated 10 times for two series, with 5 min warm-up before starting the first series, 5 min rest in between series, and 5 min of cool-down at the end of the second series (28). Patients will train with the modality that is most suitable for consistent training (either walking, running, cycling, squatting, etc.). HIIT has been already investigated in neurological populations such as stroke (17, 29). Aside from cardiovascular benefits, HIIT after skill training improves motor learning retention, likely by promoting the release of brain derived neurotrophic factors (30). Therefore, HIIT will be provided by the end of the overall training session, to both improve endurance and foster motor learning retention.

To improve motivation and adherence, dual-tasks during both resistance and motor execution training will be provided in a gamified way (31). For instance, while holding a body position to train strength or motor control, patients will play a target-reaching exercise, with lighting targets either shown at random (simple speed reaction task), shown at random but with rules according to the color of lights (choice reaction task), or according to a memory sequence of increasing difficulty (working memory task).

Each therapist-based session will last in between 60 to 120 min. The four items will be administered within each session, though variations will be allowed according to patient's needs, i.e. patients with more prominent spasticity issues will allocate more time for item #1, whereas patients with more prominent weakness or ataxia will dedicate more time on item #2. Individualization of the treatment will be defined by the treating physiotherapist at the end of the first assessment at baseline (T0), to prioritize objective impairments and patient's reported needs, capabilities, and expectations that emerged during the evaluation. At the same time, the physiotherapist will closely monitor patient's response to physical exercise to detect and report any sign of severe fatigue, over exhaustion, and symptoms related to excessive physical effort (muscle soreness, pain, cramps, dizziness, nausea, dyspnoea, etc.). To calibrate the content and intensity of training, the first two to three sessions will gradually increase the level of difficulty and physical effort, allowing sufficient time to properly instruct the patient, and to receive feedback on the physical response to exercise.

### Outcomes

The primary outcome is feasibility, in terms of recruitment, adherence, retention, safety, and patient's satisfaction. The criteria to be met for feasibility are as follows: for recruitment, 20 patients should be enrolled within 24 months since the start of the trial (September 2024). For adherence, at least 75% of planned sessions should be performed. For retention, at least 75% of patients enrolled in the study should complete the intervention (with adequate adherence). For safety, the criteria are the absence of any serious adverse event attributable to the intervention. For the present study, serious adverse event is defined not only as undesirable and unintended events requiring hospitalization, but also any event requiring medical *attention*, such as muscle strain, falls, dyspnoea, etc. For patient's satisfaction, a 5-point Likert questionnaire will quantify satisfaction of the overall treatment received, as well as for each FRAME item separately. Similarly, patient's will be asked to quantify the level of enjoyment of the treatment(s) received, how easy was for them to perform, how much was perceived useful to improve their condition, how likely it will be to implement some exercises in their weekly routine. The questionnaire will end with open questions, to ask for feedback about strengths and weaknesses of the treatment received, and social/environmental barriers and facilitators to implement such exercises in their routine. Notably, a similar questionnaire will be provided at the end of a subsequent period of 12 weeks of training at home, asking about satisfaction and perceived utility of self-administered sessions, to compare patient's perspective of therapist-guided training vs. home-based training.

The main secondary outcome will be gains in endurance as measured with the 6 Minute Walk Test (32). According to the original instructions, patients are instructed to walk along a straight 25-meter path, with visible turning points at the beginning and the end, with the goal of covering the longest distance possible within 6 min. Patients are allowed to walk independently or with the use of assistive devices, and to take rests while standing. Running, sitting, or receiving physical assistance from the therapist (other than help for balance) is not allowed. Clinically relevant changes are defined as gains of at least 15 m in the distance covered during the test (33).

Other secondary outcome measures will investigate body functions that may contribute to gains in gait capacity, such as improvements in speed, strength, mobility, muscle tone, and balance:

The Spastic Paraplegia Rating Scale (SPRS) measures disease severity and progression, by assigning a score between 0 (normal) and 4 (severe impairment) for 13 items evaluating gait (pattern, endurance, speed), stair climbing (speed and need of support), raising from a chair (speed and need of support), spasticity (hip adductor and knee flexors), weakness (hip abductors and ankle dorsiflexors), presence of lower limb contractures limiting mobility, pain associated with spastic paraparesis, and bowel and bladder function (34). In addition to traditional SPRS, a recently developed modified-SPRS (m-SPRS) will be assessed. Considering that m-SPRS provides additional qualitative and quantitative information for each item, and given the interventional nature of the planned study, the main focus will be on investigating sensitivity to change.

10 Meter Walking Test assesses walking speed (35). The setting is even floor with markers at 0, 2, 8, and 10 m. The patient is instructed to walk from the 0 to 10 m marker. Two trials are performed at ‘comfortable speed' and at ‘maximum speed'. The assessor measures the time taken to walk from 2 to 8 m marker, averaging the time of the two trials for each condition as the final score.

The Five Times Sit-To-Stand test assesses functional lower extremity strength, by measuring the time to stand up and sit down five consecutive times (36). Patients are instructed to perform the task without arm support, as fast as possible.

The Functional Reach Test assesses dynamic balance while standing (37). The patient is instructed to stand with one shoulder flexed at 90 degrees, fist closed, and to bend forward as far as possible without taking a step. Patients are standing close to a ruler taped to the wall and are not allowed to touch the wall nor to rotate the trunk while bending. The assessor measures the distance (centimeters) between the start and end position by using the third metacarpal as reference. Three attempts are performed and the average of the last two taken as the final score.

Isometric strength will be evaluated at the level of ankle dorsiflexors, ankle plantarflexors, knee extensors, knee flexors, hip extensors, hip flexors, hip abductors.

A goniometer will be used to measure the passive range of motion for ankle dorsiflexion (with knee flexed), knee flexion (with hip extended), hip abduction and hip extension (38, 39).

A stabilometric platform (Prokin 252, TecnoBody s.r.l., Bergamo, Italy) will measure static balance by detecting the center of pressure while standing. Outcomes that will be considered include the area and perimeter of the center of pressure while standing with eyes open and eyes closed, and limits of stability in antero-posterior direction.

The recently validated italian version of the ‘Hereditary Spastic Paraplegia—Self Notion and Perception Questionnaire' (HSP-SNAP) will be administered (40). HSP-SNAP assesses perceived disease-specific motor deficits affecting flexibility, strength, balance, endurance, fatigue, and pain.

To quantify the content of therapy provided, a logbook will be recorded to indicate the number of minutes per session dedicated to each item of the FRAME protocol. Within each item specific elements will be recorded as well, such as spastic muscles that received either stretching and/or electrical stimulation, weak muscles that were targeted for strength training, specific motor control exercises, type of aerobic exercise performed and rate of perceived exertion in the middle and at the end of the protocol.

### Study timeline

A convenient sample of 20 patients will be enrolled in the study over a 24-month period, starting from September 2024. Sample size has been considered adequate to determine the overall feasibility of the intervention, and informative to guide the design of larger, controlled trials in the future (41). Patients will be recruited among those present in an internal registry of patients receiving routinary healthcare services from the same institution.

**Figure 2:**
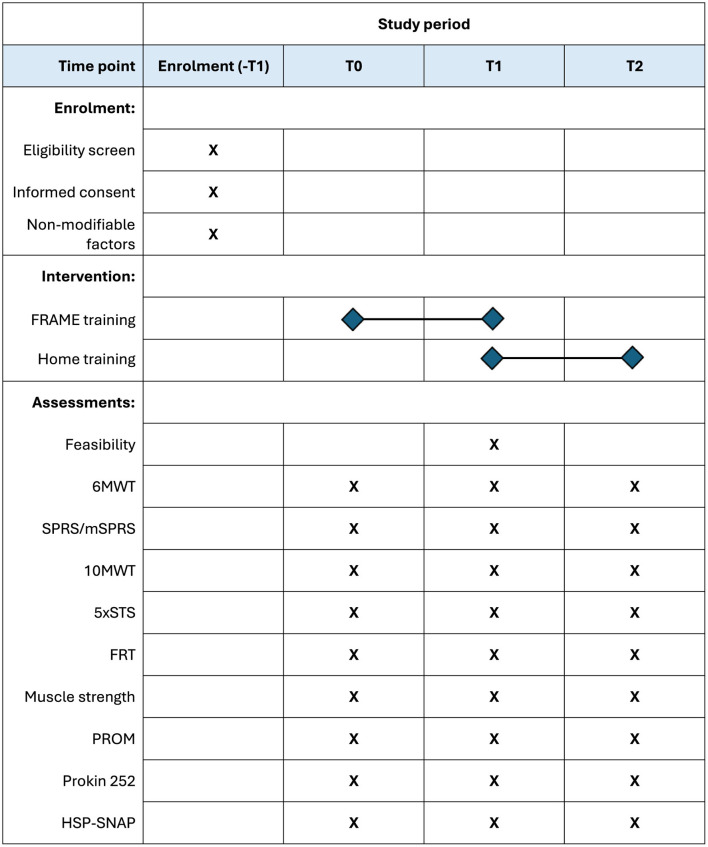
Schedule of enrolment, interventions, and assessments. Schedule of enrolment, intervention, and assessments. Upon enrolment, eligibility and informed consent will be administered, as well as reporting non-modifiable factors (age at the time of enrolment, gender, diagnosis, etc.). The intervention will last in between five to 10 weeks, according to planned number and frequency of sessions. Feasibility will be measured only at T1, while all secondary outcomes will be assessed at each time point. 10MWT, Ten-Meter Walk Test; 5xSTS, Five Times Sit-to-Stand Test; 6MWT, Six-Minute Walk Test; FRAME, Flexibility, Resistance, Aerobic, Movement Execution; FRT, Functional Reach Test; HSP-SNAP, Hereditary Spastic Paraplegia-Self Notion and Perception Questionnaire; PROM, Patient-Reported Outcome Measures; SPRS/mSPRS, Spastic Paraplegia Rating Scale/modified Spastic Paraplegia Rating Scale.

Figure 2 illustrates the study timeline. Patients will be assessed at T0 (baseline, before the intervention), T1 (5–10 weeks after T0, at the end of the intervention), and T2 (12 weeks after T1). By the time of enrolment, patients should agree to attend at least 10 sessions of training, at least once a week. Attendance of two sessions per week and for up to 16 sessions in total will be encouraged. During the intervention period, patients will be encouraged to repeat exercises at home and to keep as active as possible, and concomitant care will be allowed. By the end of the intervention, patients will receive instructions to continue exercising at home and be encouraged to keep an active lifestyle to maintain long-term benefits.

## Methods: data collection, management, and analysis

Research data will be gathered and administered through REDCap electronic data capture tools hosted at IRCCS Eugenio Medea Conegliano (42, 43). Each patient will be assigned a unique alphanumeric code to protect their identity. Access to these data files will be restricted to researchers involved in data management, authorized solely by the principal investigator. Paper-based materials, including informed consent forms, are securely kept in a locked closet at the same institution, accessible only to the principal investigator. A copy of the case report form is available as Supplementary material. Anonymized data supporting the study's conclusions can be obtained from the corresponding author upon reasonable request.

### Statistical analysis

R software will be used for both statistical analysis and graphical representation (44). Due to the limited sample size and the utilization of ordinal scales, non-parametric statistical methods are employed. Descriptively, continuous/ordinal variables are represented using median and interquartile range (IQR) to denote central tendency and dispersion, respectively. Frequencies are presented as absolute values followed by relative values (percentage) in parentheses. Binary variables, such as gender, are reported with only one of the two variables indicated. Treatment effect estimates are reported alongside actual significance levels (two-sided *p*-value) and 95% confidence intervals. Wilcoxon signed-rank test is used for quantitative and ordinal variables, while McNemar's test is applied for dichotomous variables. Kendall's tau rank correlation coefficient is utilized for correlations between quantitative/ordinal variables measured concurrently.

## Limitations

The present protocol aims at investigating whether intensive and comprehensive neurorehabilitation training is feasible and effective in patients with HSP. To determine whether the intervention is applicable to HSP patients in general, eligibility criteria allow the inclusion of patients with potentially heterogeneous genetic diagnoses, broad levels of motor impairments, and variable age and disease duration. Results from such heterogeneous cohort will be informative for feasibility but inevitably difficult to interpret for effectiveness. Another limitation is the flexibility of the intervention, that sets a bare minimum of 10 sessions but then allows patients to choose whether to perform up to 16 sessions in total, one to two sessions per week, 60–120 min of duration. Again, the heterogeneity of the dose and intensity of training will be informative of patients' preferences and capabilities, and even allow analysis of dose-response relationships, but will limit group interpretation of effectiveness for a specific amount of therapy. Moreover, the possibility to allocate more or less time to each item of the intervention, based on patient's specific needs, is a valuable opportunity to personalize treatment and possibly improve functional gains, but increase the variability of the intervention. To mitigate this factor, exploratory analyses of secondary outcomes will focus on results from the 6 Minute Walk Test, given that any improvement in flexibility, resistance, motor control, and aerobic capacity may ultimately lead to gains in gait endurance. Finally, the limited number of patients recruited for the present study will not permit full representation of all genetic forms of HSP, allowing inferences only for major subtypes.

## Data Availability

Results, as well as the methodology supporting the overall conduct of the study, will be published as peer-reviewed articles in scientific journals and presented at scientific conferences. The raw, anonymized data supporting the conclusions of this research trial will be made available by the authors, upon reasonable request.
