# Supplementary material for: Flexibility, Resistance, Aerobic, Movement Execution (FRAME) training program to improve gait capacity in adults with Hereditary Spastic Paraplegia: protocol for a single-cohort feasibility trial
Source: Front Neurol. 2025 Feb 18;16:1441512. doi: 10.3389/fneur.2025.1441512 (PMC11877901; doi:10.3389/fneur.2025.1441512)
Supplement: Supplementary file 1 [file Data_Sheet_1.pdf]

reclutamento

Record ID ______________________

hash ______________________

Data Reclutamento ______________________

Inserted by ______________________

Data di nascita ______________________

Sesso ______________________

Inclusion criteria

DATE	__________________
Specific HSP diagnosis	__________________
Age at symptoms onset	__________________ (Approximate age (years) at symptoms onset. If unknown write '100')
Duration of the disease (since symptoms onset)	__________________ (Approximate years of disease since symptoms onset. If unknown write '100')
Current pharmacotherapy	__________________ (if no drug, write '0')

Below a list of six inclusion criteria. Six "YES" are mandatory to consider the patient eligible for the study. Failure of one or more inclusion criteria results in patient exclusion.

The patient is adult (18 years or older)	◯ Yes ◯ No
Presence of any lower limb deficit affecting gait	◯ Yes ◯ No (muscle weakness, spasticity, or balance deficits)
Ability to walk without physical assistance of another person	◯ Yes ◯ No
Able to understand simple instructions, understand the purpose of the intervention, willing to participate and agree to undertake AT LEAST 10 TREATMENT SESSIONS, and able to provide informed consent.	◯ Yes ◯ No
Patient did not received botulinum toxin or surgery to remedy spasticity in the six months before enrolment	◯ Yes ◯ No (YES = no botox/surgery in the 6 months before enrolment)
ABSENCE OF CONTRAINDICATIONS to stretching, resistance training, and/or aerobic exercise such as severe musculoskeletal conditions, decompensated heart failure, severe aortic stenosis, uncontrolled arrhythmia, and acute coronary syndromes	◯ Yes ◯ No (YES = no contraindications)

FINAL DECISION

FINAL DECISION: IS THE PATIENT ELIGIBLE? ◯ Yes ◯ No

Contraindications to electrical stimulation over lower limbs:
- presence of pacemaker or any implanted electronic device
- over areas of known or suspected malignancy
- history of seizures
- pregnancy
- recently radiated tissue
- tissue infection, wounds
- recent surgery, bone fracture, osteoporosis
- active deep vein thrombosis or thrombophlebitis
- impaired circulation

The presence of contraindications is not a reason to exclude the patient, BUT it is a reason to NOT PERFORM electrical stimulation.

ABSENCE OF CONTRAINDICATIONS to electrical stimulation over lower limbs? ◯ Yes ◯ No
(YES = no contraindications)

NOTES (issues, adverse events, etc.)

____
(if nothing relevant to note, write "0")

Calendar

DATE	______________
Session 1	______________
Session 2	______________
Session 3	______________
Session 4	______________
Session 5	______________
Session 6	______________
Session 7	______________
Session 8	______________
Session 9	______________
Session 10	______________

PLANNED CALENDAR (EXTRA SESSIONS)

Session 11	______________
Session 12	______________
Session 13	______________
Session 14	______________
Session 15	______________

Session 16

____

PLANNED EVALUATIONS (T0, T1, T2)

Evaluation pre-treatment (T0)

____
(immediately before the first treatment (same week))

Evaluation post-treatment (T0)

____
(immediately after the last treatment (same week))

Evaluation follow-up (T2)

____
(12 weeks after T1)

NOTES

____

HSP_SNAP

DATE

____

Questionario Self-Notion and Perception nella Paraparesi Spastica Ereditaria (HSP-SNAP)

Scelga un punteggio per ogni affermazione.

NELL'ULTIMA SETTIMANA:

	Fortemente in disaccordo	In disaccordo	Neutrale	D'accordo	Totalmente d'accordo
1. La rigidità delle gambe ha influenzato il mio cammino.	○	○	○	○	○
2. Ho svolto le mie attività motorie quotidiane senza fatica fisica.	○	○	○	○	○
3. La debolezza delle gambe ha limitato il mio cammino.	○	○	○	○	○
4. Ho avuto una buona resistenza nel cammino.	○	○	○	○	○
5. Il dolore fisico mi ha ostacolato nelle mie attività motorie quotidiane.	○	○	○	○	○
6. Mi sono sentito fluido/sciolto nel cammino.	○	○	○	○	○
7. La scarsa resistenza nel cammino mi ha ostacolato.	○	○	○	○	○
8. Ho avuto le gambe forti nel cammino.	○	○	○	○	○
9. Ho avuto scarso equilibrio nel cammino.	○	○	○	○	○
10. Ho eseguito le mie attività motorie quotidiane senza dolore fisico.	○	○	○	○	○
11. La fatica fisica ha limitato le mie attività motorie quotidiane.	○	○	○	○	○
12. Ho avuto buon equilibrio nel cammino.	○	○	○	○	○

NOTES (issues, adverse events, etc.)

____
(if nothing relevant happened, write "0")

6MWT: 6 Minute Walk Test (endurance)

Instructions to the patient in sitting:
"The aim of this test is to walk as far as possible in six minutes. You will walk back and forth in the hallway.
Six minutes is a long time to walk, so you will be exerting yourself. You may get out of breath or become tired.
You are allowed to slow down, to stop, and to rest as necessary. You may stand and rest, but resume walking as soon as you are able.
Are you ready to do that?"

Further instructions to the patient:
"Walk to the turnaround point at each end. I am going to use this counter to keep track of the laps you complete. Remember the aim is to walk as far as possible, but do not run or jog."
"Start now or when you are ready."

Instruction to the examiner:
Encouragement (eg, "You're doing a good job and you have 5 minutes left, or "Keep up the good work. You have 4 minutes to go.") is given after each minute of the test; no other communication should occur during the test.
Patients may use any assistive device or bracing that they are currently using. The type of device and/or bracing must be documented.
When administering the test, do not walk in front of or directly beside the patient, as this may "pace" the patient and influence the speed and distance they walk. Instead, walk at least a half step behind the patient.
If a patient requires assistance, only the minimum amount of assistance required for a patient to complete the task should be provided. The level of assistance documented, however, should reflect the greatest amount of assistance provided during the test. For example, if a patient required minimum assistance for the majority of the test but required moderate assistance for stability on one occasion, the patient should be rated as requiring moderate assistance. Assistance should be provided to prevent a fall or collapsing (i.e. knee buckling, trunk collapse, etc).

DATE ______________________

Physical assistance required (by the therapist)	◯ total assistance [patient performs 0%-24% of task] IF SO, score of 6MWT is ZERO<br>◯ maximum assistance [patient performs 25%-49% of task]<br>◯ moderate assistance [patient performs 50%-74% of task]<br>◯ minimum assistance [patient performs 75%-99% of task]<br>◯ supervision [patient requires stand-by or set-up assistance; no physical contact is provided]<br>◯ modified independent [patient requires use of ASSISTIVE DEVICES or bracing, needs extra time, mild safety issues]<br>◯ INDEPENDENT<br>(assistance should not propel forward (only assistance for balance issues))
Walking aid(s) used for the test	◯ None<br>◯ Ankle-foot orthoses<br>◯ One-sided crutch or walking stick<br>◯ Bilateral crutches or walking sticks<br>◯ Walker<br>◯ Gait trainer<br>◯ Other
Total distance (meters)	__________________________________
Total time of the test (seconds)	__________________________________<br>(if completed the test: 360)
Speed	__________________________________<br>(meters/second)
NOTES (issues, adverse events, etc.)	__________________________________<br>(if nothing relevant happened, write "0")

mSPRS: modified Spastic Paraplegia Rating Scale (disease severity and progression)

DATE ______________________

ITEM 1: Walking distance without pause

Due to history. The maximum walking distance without pause that the patient feels comfortable covering on a good day on a convenient surface should be reported. Ask the patient to consider the PAST 4 WEEKS for reference. Try to give everyday life examples for a 500 m distance. Support from walking aids or an assisting person is allowed.

ITEM 1: Walking distance without pause

- ◯ 0 = Normal, unlimited compared to age group
- ◯ 1 = Abnormal exhaustion due to the neurological disease after more than 500 m
- ◯ 2 = Walking distance between 10 m and 500 m
- ◯ 3 = Walking distance less than 10 m
- ◯ 4 = Unable to walk even with support (walking aid, one assisting person or combination of both)

Additional information: ABSOLUTE DISTANCE

______________________ (meters)

Additional information: INFORMATION PROVIDED BY

- ◯ patient
- ◯ caregiver

Additional information: are there factors OTHER THAN MOTOR DYSFUNCTION related to the neurological disease that may have influenced the answer?

- ◯ YES
- ◯ NO

ITEM 2: Gait quality and maximum gait speed

Patient is asked to cover a 10 m distance as fast as possible ("Move as fast as you can without jeopardizing your safety; consider running if possible").

The distance includes one turn: 5 m walk/run straight - 180° turn - 5 m walk/run back.
If patients use a walking aid/orthosis on a regular basis in everyday life, they should use the same aid/orthosis when completing this task. Patients should wear comfortable shoes.

Clearly mark the starting line as well as the turning line on the floor. Use a stopwatch to record the time until the first foot crosses the finish line (one decimal).

Evaluate gait quality and speed in the same run of this test.

ITEM 2: Gait quality	◯ 0 = Normal<br>◯ 1 = Mildly abnormal, running still possible<br>◯ 2 = Clearly abnormal, walking without support, running not possible<br>◯ 3 = Gait abnormality requiring support<br>◯ 4 = Unable to walk for a 10 m distance even with support (walking aid, one assisting person or combination of both)

ITEM 3: Speed

ITEM 3: SCORE	◯ 0 = Normal<br>◯ 1 = Slightly reduced (10 m: ≥ 5 s)<br>◯ 2 = Moderately reduced (10 m: ≥ 10 s)<br>◯ 3 = Severely reduced (10 m: ≥ 20 s)<br>◯ 4 = Unable to walk for a 10 m distance or time ≥ 40 s<br>(Seconds to walk 10 meters)
Additional information: ABSOLUTE TIME	______________________________<br>(seconds)
Walking aid(s) used for the test	◯ None<br>◯ Ankle-foot orthoses<br>◯ One-sided crutch or walking stick<br>◯ Bilateral crutches or walking sticks<br>◯ Walker<br>◯ Gait trainer<br>◯ Other
Are there factors other than motor dysfunction related to the neurological disease that may have influenced the answer?	◯ YES<br>◯ NO

ITEM 4: Quality of stair climbing

Ask the patient to walk up 5 steps, turn around, and then walk back down at a comfortable self-selected speed.

Ask the patient to USE THE HANDRAIL AS LITTLE AS POSSIBLE as long as he/she feels safe to do so. Small children unable to reach the handrail may be accompanied by a person for light support; score 1 or 2 in this case, depending on the level of support.

The direction can be reversed (first walk down - turn - walk back up). If the patient ascends or descends sideways, backwards or touches the steps with his/her hands (e.g., crawling), score 3.

ITEM 4: Quality of stair climbing	◯ 0 = Normal: needs no support of the handrail<br>◯ 1 = Mild impairment: needs intermittent support of the handrail<br>◯ 2 = Moderate impairment: needs permanent support of the handrail<br>◯ 3 = Severe impairment: needs support of another person or additional walking aid to perform task<br>◯ 4 = Unable to climb stairs even with support
Are there factors other than motor dysfunction related to the neurological disease that may have influenced the answer?	◯ YES<br>◯ NO

ITEM 5: Speed of stair climbing

Ask the patient to walk up 5 steps, turn around, and then walk back down as fast as possible ("Go up and down the stairs as fast as you can without risking a fall").

Please advise the patient that he/she should USE THE HANDRAIL TO ALLOW BETTER STANDARDIZATION OF THE TASK. Small children unable to reach the handrail may be accompanied by a person for light support.

The direction can be reversed (first walk down - turn - walk back up). Please clearly mark the turning point on the stairs. Use a stopwatch to record the time it takes for both feet to reach the last step (one decimal place).

ITEM 5: Speed of stair climbing	◯ 0 = Normal<br>◯ 1 = Slightly reduced (≥ 5 s to perform task)<br>◯ 2 = Moderately reduced (≥ 10 s to perform task)<br>◯ 3 = Severely reduced (≥ 20 s to perform task)<br>◯ 4 = Unable to climb stairs even with support
Additional information: ABSOLUTE TIME	______________________________<br>(seconds)
Are there factors other than motor dysfunction related to the neurological disease that may have influenced the answer?	◯ YES<br>◯ NO

ITEM 6: Arising from chair

Have the patient sit in a straight-backed chair with armrests, with both feet on the floor and sitting back in the chair (chair needs to be appropriate to the size of the patient).

Ask the patient to cross the arms in front of the chest and then to stand up.
If the patient is not successful, repeat this attempt up to a maximum of two more times.
If still unsuccessful, allow the patient to move forward in the chair to arise with arms folded across the chest. Allow only one attempt in this situation.
If unsuccessful, allow the patient to push off using the hands on the armrests. Allow a maximum of three trials of pushing off.
If the patient needs assistance by an additional person, walking aid or other item (e.g., table) to arise, score 4.

ITEM 6: Arising from chair	◯ 0 = Normal<br>◯ 1 = Slow, may need more than one attempt or may need to move forward in the chair to arise. No need to use the armrests<br>◯ 2 = Pushes self-up using his/her hands on the armrests, but can get up without difficulty.<br>◯ 3 = Needs to push off, but tends to fall back; or may have to try more than one time using the arms of the chair but can get up without help.<br>◯ 4 = Unable to arise without help.
Are there factors other than motor dysfunction related to the neurological disease that may have influenced the rating?	◯ YES<br>◯ NO

ITEM 7: Spasticity - hip adductor muscles (Modified Ashworth scale)

Test in supine position. Stand at the end of the examination table and lift both of the patient's legs at the ankles.

Ask the patient to relax his/her legs.
Starting from a maximally adducted position with extended knees, slowly move the legs apart to determine the passive range of motion.
Then, return to the adducted position and quickly move the legs apart.
Return to the starting position and repeat the rapid outward movement two more times.

To evaluate spasticity, consider the increased muscle tone during rapid movements within the individual range of motion of the patient.
The more severely affected side determines the rating.

ITEM 7: Spasticity - hip adductor muscles (Modified Ashworth scale)	◯ 0 = No increase in muscle tone<br>◯ 1 = Slight increase in muscle tone, manifested by a catch and release<br>◯ 2 = More marked increase in muscle tone through most of the range of motion, but affected part(s) easily moved<br>◯ 3 = Considerable increase in muscle tone - passive movement is difficult<br>◯ 4 = Limb stiff in adduction
Use of antispastic medication?	◯ YES<br>◯ NO
Are there factors other than motor dysfunction related to the neurological disease that may have influenced the rating?	◯ YES<br>◯ NO

ITEM 8: Spasticity - knee extensor muscles (Modified Ashworth scale)

Test in supine position. Start with one leg, making sure it is relaxed.

Place your hands from both sides under the knee joint and quickly pull up the knee, inducing a rapid knee flexion. Presence of a 'catch' will result in the patient's heel briefly leaving the surface of the examination table during this maneuver (score at least 1).

Then, lift the leg, holding it by the ankle and the knee joint in a maximally extended position. Ask the patient to relax the leg. Rapidly move the leg to a position of maximum knee flexion. Return to the extended position and repeat the rapid flexion movement two more times. Evaluate spasticity during the rapid flexion movement. Perform the test on the other leg. The more severely affected side determines the rating.

ITEM 8: Spasticity - knee extensor muscles (Modified Ashworth scale)	◯ 0 = No increase in muscle tone<br>◯ 1 = Slight increase in muscle tone, manifested by a catch and release<br>◯ 2 = More marked increase in muscle tone through most of the range of motion, but affected part(s) easily moved<br>◯ 3 = Considerable increase in muscle tone - passive movement is difficult<br>◯ 4 = Limb stiff in adduction
Use of antispastic medication?	◯ YES<br>◯ NO
Are there factors other than motor dysfunction related to the neurological disease that may have influenced the rating?	◯ YES<br>◯ NO

ITEM 9: Weakness - hip abduction (Medical Research Council 1976)

Test in supine position. Ask the patient to abduct the legs in an extended position. Place your hands at the ankles.

Try to push the patient's feet together while asking the patient to hold the legs in the abducted position. Make sure to slowly increase the force you apply as this item is intended to measure maximum strength and not fast recruitment.

If the range of motion is limited, rate strength in the remaining range of motion.
If weakness is present, ask the patient to turn to the side and raise the leg that is now laying on top in an extended position to evaluate the exact Medical Research Council (MRC) grade.
The more severely affected side determines the rating.

ITEM 9: Weakness - hip abduction (Medical Research Council 1976)	◯ 0 = No weakness<br>◯ 1 = Mild weakness (4/5)<br>◯ 2 = Moderate weakness (3/5)<br>◯ 3 = Severe weakness (1-2/5)<br>◯ 4 = Plegia (0/5)
Are there factors other than motor dysfunction related to the neurological disease that may have influenced the rating?	◯ YES<br>◯ NO

ITEM 10: Weakness - foot dorsiflexion (Medical Research Council 1976)

Test in supine position. Ask the patient to dorsiflex the feet ('pull your toes to your nose') one at a time and hold the foot in a dorsiflexed position.

Place your hands on the ankle and the forefoot and try to push the foot down. Make sure to slowly increase the force you apply as this item is intended to measure maximum strength and not fast recruitment. Perform the test on the other leg.

If contractures are present limiting the range of motion, rate strength in the available range of motion.
If the joint is immobile (ankylosis), score 4.
The more severely affected side determines the rating.

ITEM 10: Weakness - foot dorsiflexion (Medical Research Council 1976)	◯ 0 = No weakness<br>◯ 1 = Mild weakness (4/5)<br>◯ 2 = Moderate weakness (3/5)<br>◯ 3 = Severe weakness (1-2/5)<br>◯ 4 = Plegia (0/5)
Reduced range of motion?	◯ YES<br>◯ NO
Ankylosis present?	◯ YES<br>◯ NO
Are there factors other than motor dysfunction related to the neurological disease that may have influenced the rating?	◯ YES<br>◯ NO

ITEM 11: Contractures of lower limbs

Test in supine position moving the joint as slowly as possible to assess its range of motion (by visual inspection without using a goniometer).

- Hip joint contractures: positive Thomas test (hip flexors) or maximum angle between abducted legs ≤ 60° (hip
adductors, test with extended leg).

- Knee joint contracture: knee cannot be fully extended or flexed.

- Ankle joint contracture (test with extended leg): dorsiflexion of the foot ≤ 10° or pronation of

the foot ≤ 10°.

Rate contractures across the three joint levels: hip - knee - ankle.

ITEM 11: Contractures of lower limbs	◯ 0 = Normal<br>◯ 1 = Slight resistance throughout the course of slow passive movement of one or more joints<br>◯ 2 = Contracture of one joint level (unilaterally or bilaterally)<br>◯ 3 = Contracture of two joint levels (unilaterally or bilaterally)<br>◯ 4 = Contracture of three joint levels (unilaterally or bilaterally)
Are there factors other than motor dysfunction related to the neurological disease that may have influenced the rating?	◯ YES<br>◯ NO

ITEM 12: Pain due to HSP-related symptoms

How severely has pain due to spastic paraplegia related symptoms impacted the everyday life OVER THE PAST 4 WEEKS.
Enter your clinical assessment here, based on an exploration of the severity of the pain in a dialog with the patient.

ITEM 12: Pain due to HSP-related symptoms	◯ 0 = No pain<br>◯ 1 = Mild pain (may be bothersome, but can be ignored most of the time)<br>◯ 2 = Moderate (constantly aware of pain, but can continue most activities)<br>◯ 3 = Distressing (think of pain all of the time, give up many activities because of pain)<br>◯ 4 = Severe pain (hard to think of anything else but pain, can barely talk or move because of the pain)
Information provided by	◯ Patient<br>◯ Relative or caregiver
Regular use of analgesic medication?	◯ YES<br>◯ NO

ITEM 13: Bladder function

Rate this item considering symptoms OVER THE PAST 4 WEEKS.

Definitions:
- Low volume leakage/incontinence: patient loses small volumes of urine, typically requiring use of protective
pads.
- High volume leakage/incontinence: patient loses large volumes of urine, typically requiring use of incontinence
pants/briefs/diaper or change of clothing after the incident.
- Do not consider singular instances of incontinence, when caused by exceptional circumstances (e.g., bladder infection, long trip) in the rating.

ITEM 13: Bladder function	◯ 1 = Urgency without unwanted loss of urine or mild hesitance (e.g., takes longer than usual to empty bladder)<br>◯ 2 = Urgency with low volume leakage/incontinence or moderate hesitance (e.g., using auxiliary voiding<br>◯ repeated attempts to empty bladder or equivalent)<br>◯ 3 = High volume leakage/incontinence or severe hesitance (requiring considerable effort to empty the bladder, severely impacting everyday life)<br>◯ 4 = Severely impaired bladder control requiring permanent pants/briefs/diaper or daily intermittent/permanent catheterization
Information provided by	◯ Patient<br>◯ Relative or caregiver
Use of medication for bladder control?	◯ YES<br>◯ NO
TOTAL mSPRS score	__________________________________
NOTES (issues, adverse events, etc.)	__________________________________<br>(if nothing relevant happened, write "0")

FRT: Functional Reach Test (balance)

Instructions:

The patient is instructed to next to, but not touching, a wall and position the arm that is closer to the wall at 90 degrees of shoulder flexion with a closed fist. The patient cannot turn the trunk while bending forward.

The assessor records the starting position at the 3rd metacarpal head on the yardstick.

Instruct the patient to "Reach as far as you can forward without taking a step."

The location of the 3rd metacarpal is recorded. Scores are determined by assessing the difference between the start and end position is the reach distance.

Three trials are done and the average of the last two is noted.

A yardstick and duck tap will be needed for the assessment. The yardstick should be affixed to the wall at the level of the patient's acromion.

DATE

____

Trial 1

____
(centimeters)

Trial 2

____
(centimeters)

Trial 3

____
(centimeters)

FRT score

____

NOTES (issues, adverse events, etc.)

____
(if nothing relevant happened, write "0")

Prokin 252 (balance)

Perform the test with standardized feet placement, and with the trunk sensor.

Perform only one trial with eyes open, and only one trial with eyes closed.

Perform three trials with limits of stability, and take note of all scores. The final score is the average of the last two trials.

DATE ______________________

Stabilometry EYES OPEN

Area ______________________ (mm2)

Perimeter ______________________ (mm)

Standard deviation ANTERIOR-POSTERIOR ______________________ (mm)

Standard deviation MEDIO-LATERAL ______________________ (mm)

Average COP (Y) ______________________ (mm)

Average COP (X) ______________________ (mm)

Angle regression line ______________________ (mm)

Standard deviation TRUNK ______________________ (mm)

Average speed Anterior-Posterior ______________________ (mm/s)

Average speed Medio-Lateral

________________ (mm/s)

Stabilometry EYES CLOSED

Area

________________ (mm2)

Perimeter

________________ (mm)

Standard deviation ANTERIOR-POSTERIOR

________________ (mm)

Standard deviation MEDIO-LATERAL

________________ (mm)

Average COP (Y)

________________ (mm)

Average COP (X)

________________ (mm)

Angle regression line

________________ (mm)

Standard deviation TRUNK

________________ (mm)

Average speed Anterior-Posterior

________________ (mm/s)

Average speed Medio-Lateral

________________ (mm/s)

ROMBERG TEST

ROMBERG TEST

Area EC/EO ____________________

ROMBERG TEST

Perimeter EC/EO ____________________

Limit of stability - TRIAL 1

Anterior limit ____________________ (%)

Posterior limit ____________________ (%)

Right side limit ____________________ (%)

Left side limit ____________________ (%)

Anterior-Right side limit ____________________ (%)

Anterior-left side limit ____________________ (%)

Posterior-right side limit ____________________ (%)

Posterior-left side limit ____________________ (%)

Limit of stability - TRIAL 2

Anterior limit ____________________ (%)

Posterior limit ____________________ (%)

Right side limit

_______________________ (%)

Left side limit

_______________________ (%)

Anterior-Right side limit

_______________________ (%)

Anterior-left side limit

_______________________ (%)

Posterior-right side limit

_______________________ (%)

Posterior-left side limit

_______________________ (%)

Limit of stability - TRIAL 3

Anterior limit

_______________________ (%)

Posterior limit

_______________________ (%)

Right side limit

_______________________ (%)

Left side limit

_______________________ (%)

Anterior-right side limit

_______________________ (%)

Anterior-left side limit

_______________________ (%)

Posterior-right side limit

_______________________ (%)

Posterior-left side limit

____
(%)

FINAL SCORES (average of the last two trials)

Final score - Anterior limit

____

Final score - Posterior limit

____

Final score - Right side limit

____

Final score - Left side limit

____

Final score - Anterior-right side limit

____

Final score - Anterior-left side limit

____

Final score - Posterior-right side limit

____

Final score - Posterior-left side limit

____

NOTES (issues, adverse events, etc.)

____
(if nothing relevant happened, write "0")

10MWT: 10 Metre Walking Test (speed)

OVERVIEW: The 10MWT is used to assess walking speed in meters/second (m/s) over a short distance.

SCORING: The total time taken to AMBULATE 6 METERS (m) is recorded to the nearest hundredth of a second. 6 m is then divided by the total time (in seconds) taken to ambulate and recorded in m/s

SET-UP: Measure and mark the start and end point of a 10-m walkway. Add a mark at 2 m and 8 m (identifying the CENTRAL 6 METERS WHICH WILL BE TIMED).

The time is started when any part of the leading foot crosses the plane of the 2-m mark. The time is stopped when any part of the leading foot crosses the plane of the 8-m mark.

DATE	______________________
Physical assistance required (by the therapist)	◯ total assistance [patient performs 0%-24% of task] IF SO, score of 6MWT is ZERO<br>◯ maximum assistance [patient performs 25%-49% of task]<br>◯ moderate assistance [patient performs 50%-74% of task]<br>◯ minimum assistance [patient performs 75%-99% of task]<br>◯ supervision [patient requires stand-by or set-up assistance; no physical contact is provided]<br>◯ modified independent [patient requires use of ASSISTIVE DEVICES or bracing, needs extra time, mild safety issues]<br>◯ INDEPENDENT<br>(assistance should not propel forward (only assistance for balance issues))
Walking aid(s) used for the test	◯ None<br>◯ Ankle-foot orthoses<br>◯ One-sided crutch or walking stick<br>◯ Bilateral crutches or walking sticks<br>◯ Walker<br>◯ Gait trainer<br>◯ Other
Time of COMFORTABLE walking speed, trial 1<br><br>Instructions to patient: "Walk at your own comfortable walking pace and stop when you reach the far mark."	______________________<br>(Time taken to walk the middle 6 meters, if unable write "0")
Time of COMFORTABLE walking speed, trial 2	______________________<br>(Time taken to walk the middle 6 meters, if unable write "0")

Time of FAST walking speed, trial 1<br><br>Instructions to patient: "Walk as fast as you can safely walk and stop when you reach the far mark."	______________________<br>(Time taken to walk the middle 6 meters, if unable write "0")
Time of FAST walking speed, trial 2	______________________<br>(Time taken to walk the middle 6 meters, if unable write "0")
COMFORTABLE WALKING SPEED	______________________<br>(average of the trials, METERS/SECONDS)
FAST WALKING SPEED	______________________<br>(average of the trials, METERS/SECONDS)
NOTES (issues, adverse events, etc.)	___________________________<br>(if nothing relevant happened, write "0")

5xSTS: 5-times Sit to Stand (strength)

Overview: The test provides a method to quantify functional lower extremity strength and/or identify movement strategies a patient uses to complete transitional movements.

Scoring: The score is the amount of time (to the nearest decimal in seconds) it takes a patient to transfer from a seated to a standing position and back to sitting five times.

Logistic:
One trial is administered.
A patient is instructed to sit with arms folded across their chest and with back against the chair. A patient with hemiplegia can have the impaired arm at his/her side or in a sling.

Instruct the patient: "I want you to stand up and sit down five times in a row, as quickly as you can, when I say 'Go'. Be sure to stand up fully and try not to let your back touch the chair back between each repetition. Do not use the back of your legs against the chair."

Time starts when the tester says "Go."
Time stops when the patient's body touches the chair following the fifth repetition.
If individuals are unable to complete the first sit to stand independently, without use of arms, the test is terminated.

DATE

____

Time to complete the test

____
(Time taken in seconds, if unable write "0")

NOTES (issues, adverse events, etc.)

____
(if nothing relevant happened, write "0")

Inclinometer (passive range of motion)

DATE ______________________

LEFT SIDE

Ankle dorsiflexion ______________________
(with knee flexed)

Knee flexion ______________________
(with hip extended)

Hip abduction ______________________
(with hip extended)

Hip extension ______________________
(side lying)

RIGHT SIDE

Ankle dorsiflexion ______________________
(with knee flexed)

Knee flexion ______________________
(with hip extended)

Hip abduction ______________________
(with hip extended)

Hip extension ______________________
(side lying)

NOTES (issues, adverse events, etc.) ______________________
(if nothing relevant happened, write "0")

Dynamometer (isometric strength)

DATE ________________

LEFT SIDE

Ankle dorsiflexors ________________

Ankle plantarflexors ________________

Knee extensors ________________

Knee flexors ________________

Hip extensors ________________

Hip flexors ________________

Hip abductors ________________

RIGHT SIDE

Ankle dorsiflexors ________________

Ankle plantarflexors ________________

Knee extensors ________________

Knee flexors ________________

Hip extensors ________________

Hip flexors ________________

Hip abductors ________________

NOTES (issues, adverse events, etc.)

____
(if nothing relevant happened, write "0")

Therapeutic plan

DATE	______________________
Main problem (motor impairment and/or activity limitation) related to the neurological disease	______________________
Intervention that the patient would likely benefit the most (i.e. related to the main problem)	◯ Flexibility<br>◯ Resistance training/core stability<br>◯ Motor execution<br>◯ Aerobic/High Intensity Interval Training
Secondary problems	______________________
Positive factors (for instance, the patient has a positive attitude/active coping strategy)	______________________
Negative factors/yellow flags/reasons for caution	______________________
NOTES	______________________

Daily Session

Record ID	________________
DATE	________________
SESSION REPORT	________________ (briefly describe training performed)

ITEM 1: FLEXIBILITY

Total time dedicated to flexibility	________________ (minutes)
Stretching: Ankle plantarflexors	◯ YES ◯ NO
Stretching: Knee flexors	◯ YES ◯ NO
Stretching: Knee extensors (vasti)	◯ YES ◯ NO
Stretching: Hip adductors	◯ YES ◯ NO
NMES: Ankle plantarflexors	◯ YES ◯ NO
NMES: Knee flexors	◯ YES ◯ NO
NMES: Knee extensors (vasti)	◯ YES ◯ NO
NMES: Hip adductors	◯ YES ◯ NO

ITEM 2: RESISTANCE TRAINING

Total time dedicated to resistance training	________________ (minutes)
Strength: Addominal muscles	◯ YES ◯ NO
Strength: Back muscles	◯ YES ◯ NO
Strength: Gluteus maximus	◯ YES ◯ NO
Strength: Medium gluteus	◯ YES ◯ NO
Strength: Quadriceps	◯ YES ◯ NO
Strength: Hamstrings	◯ YES ◯ NO

Strength: Plantarflexors	◯ YES ◯ NO
Strength: Dorsiflexors	◯ YES ◯ NO

ITEM 3: MOTOR EXECUTION (stance and gait training)

Total time dedicated to motor execution training	__________________ (minutes)
Stance training: bodyweight shift (double stance)	◯ YES ◯ NO
Stance training: monopodal stance	◯ YES ◯ NO
Stance training: double stance with heel of the forward foot on ground	◯ YES ◯ NO
Stance training: double stance with toes of the backward foot on ground	◯ YES ◯ NO
Gait speed training	◯ YES ◯ NO
Side walk training	◯ YES ◯ NO
Backward gait training	◯ YES ◯ NO
Change in direction training	◯ YES ◯ NO
Stair training	◯ YES ◯ NO
Obstacles training	◯ YES ◯ NO

ITEM 4: High Intensity Interval Training

Total time dedicated to aerobic training	__________________ (minutes)
HIIT modality	◯ HIIT not performed<br>◯ walking rapidly, running<br>◯ squatting, sit to stand<br>◯ cycling (legs)<br>◯ cycling (arms)<br>◯ swimming<br>◯ other
Total number of intervals performed	__________________ (one interval corresponds to 30 seconds all-out, 30 seconds passive rest)

Rate of perceived exertion after the first 5 intervals	◯ 6 = no exertion at all<br>◯ 7 = extremely light<br>◯ 8<br>◯ 9 = Very light<br>◯ 10<br>◯ 11 = Light<br>◯ 12<br>◯ 13 = Somewhat hard<br>◯ 14<br>◯ 15 = Hard (heavy)<br>◯ 16<br>◯ 17 = Very hard<br>◯ 18<br>◯ 19 = Extremely hard<br>◯ 20 = Maximal exertion
Rate of perceived exertion at the end of HIIT	◯ 6 = no exertion at all<br>◯ 7 = extremely light<br>◯ 8<br>◯ 9 = Very light<br>◯ 10<br>◯ 11 = Light<br>◯ 12<br>◯ 13 = Somewhat hard<br>◯ 14<br>◯ 15 = Hard (heavy)<br>◯ 16<br>◯ 17 = Very hard<br>◯ 18<br>◯ 19 = Extremely hard<br>◯ 20 = Maximal exertion

Adverse events and general notes

Any adverse event occurred during the session?	◯ Yes<br>◯ No
There was a causal relationship with the intervention?	◯ Yes<br>◯ No

Was it a SERIOUS adverse event?

Definition of serious adverse event:
- death,a life-threatening adverse event requiring inpatient hospitalization (not required as part of the treatment)
- or prolongation of existing hospitalization,
- a persistent or significant disability or incapacity,
- or cancer,
- or a congenital anomaly
- or birth defect.

Important medical events that may not result in the listed outcomes may be considered as serious when, based upon appropriate medical judgment, they represent significant hazards or potentially serious harm to the research subject or others and may require medical intervention to prevent one of the outcomes listed in this definition.

◯ Yes
◯ No

Please specify the dynamic of the adverse event (cause, nature of the event, short term remedy, long term outcomes)

____

NOTES

____
(if nothing relevant happened, write "0")

Feasibility_adherence_safety

Record ID ______________________

DATE ______________________

ADHERENCE

Number of PLANNED sessions ______________________

Number of PERFORMED sessions ______________________

Adherence ______________________

Ratio between performed and planned sessions, expressed as percentage

SAFETY

Number of adverse events occurred ______________________

Number of SERIOUS adverse events occurred ______________________

Number of adverse events occurred ATTRIBUTABLE to the intervention ______________________

Number of SERIOUS adverse events occurred ATTRIBUTABLE to the intervention ______________________

NOTES ______________________

Feasibility_satisfaction_questionnaire_ITA

Record ID ______________________

DATE ______________________

Gentile partecipante, ti chiediamo di rispondere, a parole tue e secondo il tuo punto di vista, alle domande inerenti al trattamento ricevuto.

Principali punti di forza/aspetti positivi della terapia ricevuta ______________________

Principali punti critici/aspetti negativi/da migliorare della terapia ricevuta ______________________

Fattori che potrebbero facilitare o al contrario ostacolare la prosecuzione dell'allenamento a domicilio ______________________

Potresti descrivere in UNA PAROLA la tua esperienza durante questo ciclo di terapie? ______________________

Questionario di soddisfazione del trattamento ricevuto.

Gentile partecipante, il presente questionario ha l'obbiettivo di quantificare il tuo punto di vista del trattamento ricevuto. Ti chiediamo gentilmente di leggere attentamente e di rispondere a ogni domanda selezionando una sola opzione per domanda.

Grazie!

	per niente	non tanto	abbastanza	molto	moltissimo
domanda 1: NEL COMPLESSO, quanto ti ritieni soddisfatto del trattamento ricevuto?	○	○	○	○	○
domanda 2: Quanto ti ritieni soddisfatto dell'allenamento della FLESSIBILITA'?	○	○	○	○	○
domanda 3: Quanto ti ritieni soddisfatto dell'allenamento della FORZA?	○	○	○	○	○

domanda 4: Quanto ti ritieni soddisfatto dell'allenamento della RESISTENZA?	◯	◯	◯	◯	◯
domanda 5: Quanto ti ritieni soddisfatto dell'allenamento del CONTROLLO MOTORIO?	◯	◯	◯	◯	◯
domanda 6: Quanto è stato 'divertente' o 'piacevole' sottoporsi a questo ciclo di terapie?	◯	◯	◯	◯	◯
domanda 7: Quanto è stato 'difficile', 'faticoso', 'sfidante', 'impegnativo' sottoporsi a questo ciclo di terapie?	◯	◯	◯	◯	◯
domanda 8: Quanto hai ritenuto questa terapia UTILE per migliorare la tua condizione?	◯	◯	◯	◯	◯
domanda 9: Quanto consideri RISCHIOSO per la tua salute la pratica degli esercizi proposti?	◯	◯	◯	◯	◯
domanda 10: Quanto è probabile che integrerai alcuni degli esercizi proposti nella tua vita quotidiana (sessioni di allenamento giornaliere/settimanali/mensili)?	◯	◯	◯	◯	◯

NOTE AGGIUNTIVE

(qualsiasi altro commento da parte del paziente) ________________________________

Home training_questionnaire

Record ID ______________________________

NOTE AGGIUNTIVE

(qualsiasi altro commento da parte del paziente) ______________________________

DATE ______________________________

Negli ultimi 3 mesi, quante sessioni di allenamento a domicilio hai svolto? ______________________________
(number of sessions)

Gentile partecipante, ti chiediamo di rispondere, a parole tue e secondo il tuo punto di vista, alle domande inerenti al alla terapia svolta a domicilio.

Principali punti di forza/aspetti positivi della terapia a domicilio ______________________________

Principali punti critici/aspetti negativi/da migliorare della terapia a domicilio ______________________________

Fattori che potrebbero facilitare o al contrario ostacolare la prosecuzione dell'allenamento a domicilio ______________________________

Potresti descrivere in UNA PAROLA la tua esperienza durante la prosecuzione del trattamento a domicilio? ______________________________

Questionario di soddisfazione del trattamento svolto a domicilio.

Gentile partecipante, il presente questionario ha l'obbiettivo di quantificare il tuo punto di vista del trattamento ricevuto. Ti chiediamo gentilmente di leggere attentamente e di rispondere a ogni domanda selezionando una sola opzione per domanda.

Grazie!

	per niente	non tanto	abbastanza	molto	moltissimo
domanda 1: NEL COMPLESSO, quanto ti ritieni soddisfatto del trattamento svolto?	○	○	○	○	○

domanda 2: Quanto ti ritieni soddisfatto dell'allenamento della FLESSIBILITA'?	○	○	○	○	○
domanda 3: Quanto ti ritieni soddisfatto dell'allenamento della FORZA?	○	○	○	○	○
domanda 4: Quanto ti ritieni soddisfatto dell'allenamento della RESISTENZA?	○	○	○	○	○
domanda 5: Quanto ti ritieni soddisfatto dell'allenamento del CONTROLLO MOTORIO?	○	○	○	○	○
domanda 6: Quanto è stato 'divertente' o 'piacevole' sottoporsi a questo allenamento a domicilio?	○	○	○	○	○
domanda 7: Quanto è stato 'difficile', 'faticoso', 'sfidante', 'impegnativo' sottoporsi a questo allenamento a domicilio?	○	○	○	○	○
domanda 8: Quanto hai ritenuto questa terapia UTILE per migliorare la tua condizione?	○	○	○	○	○
domanda 9: Quanto consideri RISCHIOSO per la tua salute la pratica degli esercizi proposti?	○	○	○	○	○
domanda 10: Quanto è probabile che integrerai alcuni degli esercizi proposti nella tua vita quotidiana (sessioni di allenamento giornaliere/settimanali/mensili)?	○	○	○	○	○
domanda 11: Quanto hai ritenuto utile ricevere istruzioni per iscritto?	○	○	○	○	○
domanda 12: Quanto hai ritenuto utile ricevere istruzioni attraverso video tutorial?	○	○	○	○	○
